# Fencing bodes a rapid collapse of the unique Greater Mara ecosystem

**DOI:** 10.1038/srep41450

**Published:** 2017-01-25

**Authors:** Mette Løvschal, Peder Klith Bøcher, Jeppe Pilgaard, Irene Amoke, Alice Odingo, Aggrey Thuo, Jens-Christian Svenning

**Affiliations:** 1 Department of Archaeology, Aarhus University, Højbjerg, 8270, Denmark; 2 Interacting Minds Centre, Aarhus University, Aarhus C, 8000, Denmark; 3 Section for Ecoinformatics & Biodiversity, Department of Bioscience, Aarhus University, Aarhus C, 8000, Denmark; 4 Kenya Wildlife Trust, Nairobi, P.O. Box 86-005200, Karen, Kenya; 5 Maasai Mara Wildlife Conservancies Association, P.O. Box 984-20500 Narok, Kenya; 6 Department of Geography and Environmental Studies, University of Nairobi, Nairobi, P.O. Box 30197-00100, Kenya; 7 Department of Environmental Studies, Forestry and Agriculture, Maasai Mara University, Narok, P.O. Box 861-20500, Kenya

## Abstract

With land privatization and fencing of thousands of hectares of communal grazing areas, East Africa is struggling with one of the most radical cultural and environmental changes in its history. The 668,500-hectare Greater Mara is of crucial importance for the great migrations of large mammals and for Maasai pastoralist culture. However, the magnitude and pace of these fencing processes in this area are almost completely unknown. We provide new evidence that fencing is appropriating land in this area at an unprecedented and accelerating speed and scale. By means of a mapped series of multispectral satellite imagery (1985–2016), we found that in the conservancies with the most fences, areal cover of fenced areas has increased with >20% since 2010. This has resulted in a situation where fencing is rapidly increasing across the Greater Mara, threatening to lead to the collapse of the entire ecosystem in the near future. Our results suggest that fencing is currently instantiating itself as a new permanent self-reinforcing process and is about to reach a critical point after which it is likely to amplify at an even quicker pace, incompatible with the region’s role in the great wildebeest migration, wildlife generally, as well as traditional Maasai pastoralism.

In many areas of Africa, anthropogenic pressures and changes in land use are currently causing severe fragmentation of ecosystems and wildlife habitats, directly reflected in widespread declines of large mammal populations and the loss of long-distance seasonal migrations^1^. A key cause to these trends is exponential human population growth, declining rainfall, increasing livestock numbers as well as increased agriculture, infrastructure, urban and tourism developments. An even more critical threat is the expanding use of fencing. Fencing has both costs and benefits to people and wildlife. Many fences are built to prevent poaching and illegal resource extraction as well as to anticipate human-wildlife conflicts and keep diseases from being transmitted between wildlife and livestock^2^,^3^,^4^.I fences are also embedded in social and juridical ownership statements and reflect a wish to create grass banks for use by livestock. Concurrently, however unintended, the severe and costly side effects of fencing include extensive, multi-scalar habitat deterioration and fractioning of the more natural parts of the landscape into smaller, disconnected areas such as privately enclosed land parcels, but also national reserves and conservancies which are not necessarily spatially connected^5^,^6^.

Many private fences in East Africa and veterinary fences in Southern Africa are currently causing over-stocking and rangeland degradation. To wildlife, such barriers hinder access to vital resources including water supplies and salt licks, leading to entanglements in the fence or entrapments by funneling wildlife into blind corners as well as alterations of breeding behavior and a lowering of the systems’ resilience^7^,^8^. For example, in southern Kalahari, Botswana one of the greatest wildebeest migrations in Africa was destroyed by veterinary fences, and hundreds of thousands wildebeest died during the severe droughts in 1979–1985 when trying to reach the Boteti river^9^,^10^.

Many national parks in East and Southern Africa are furthermore building electric fences to reduce human-wildlife conflict, often spanning hundreds of kilometers, without any possibility for wildlife to passage. For example, the western boundary of the Kruger National Park was fenced in 1959–1961 and, in effect, incited a new game-proof fence to be built in 1966 that cut off and redirected a wildebeest migration with directly observable negative consequences on population size^11^.

Our case concerns the Greater Mara ecosystem, located in Narok County, is home to the largest and most species-diverse mammal migration in the world, including 1.3 million wildebeests (*Connochaetes taurinus*), 200,000 zebras (*Equus burchelli*) and hundreds of thousands of Thomson’s gazelles (*Gazella thomsoni*)^12^. Massive megafauna migrations were once common all over the world, but today the Mara-Serengeti migrations are among the last of their kind, representing a unique and irreplaceable African heritage^13^,^14^,^15^. The Greater Mara ecosystem is also home to the famous Maasai tribe. The traditional Maasai get their primary food from cattle, sheep and goats. When the grazing resources grow scarce, they move their family and cattle to other parts of Narok County, to surrounding counties and to Tanzania. Hence, both the migrating large mammals and migrating tribes of Maasai are dependent on access to the common resources that the ecosystem provides.

However, owing to land privatization and the increased fencing of grass banks and cultivated fields, an incompatible land use is rapidly expanding across the region. This process threatens the freedom of movement of wildlife and livestock and forewarns the collapse of the Greater Mara ecosystem. Furthermore, the Maasai are gradually abandoning their semi-nomadic lifestyle, which is dependent on seasonal grazing by livestock and wildlife, customary land holding and the sharing of common resources. Instead, they are adopting a more sedentary lifestyle involving claiming and marking private ownership of land with fences.

Unfortunately, to date, there exists no systematic overview of the fencing situation across the Greater Mara, including its area coverage and geospatial dynamics. Consequently, little knowledge is available about the present conditions and their long-term ecological and cultural consequences. This lack of data and knowledge prevents the introduction of sustainable strategies and policies to anticipate and prevent an ecological and cultural collapse, and are the motivation for this study.

## Fencing the Greater Mara

The Greater Mara is a 668,500-hectare large area in southwestern Kenya. Maasai Mara refers to a gazetted national reserve managed by the Narok County Government situated within this area. The remaining Greater Mara comprises smaller administrative areas, including wildlife conservancies, conservation areas and settlement areas. A wildlife conservancy describes land set aside by an individual landowner, body corporate, group of owners or a community for purposes of wildlife conservation in accordance with the provisions of the Wildlife Conservation and Management Act in Maasai Mara^16^.

As long ago as 1970, land tenure was formalized across the entire Greater Mara with the establishment of group ranches which have subsequently been subject to subdivision into smaller land parcels, a process which is still ongoing in some parts of the Greater Mara^17^,^18^.

**Figure 1:**
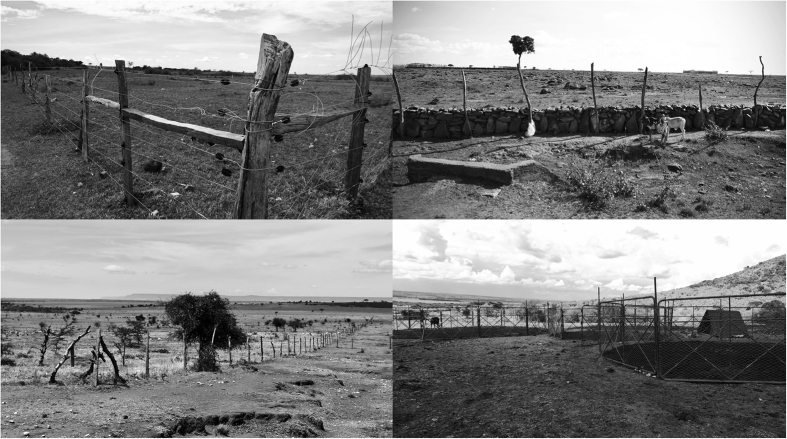
Examples of fences in the Greater Mara. By Mette Løvschal.

The fences appear in a range of different constructions, the majority of which are enclosures resulting from the enclosure of private land plots (Fig. 1). In the Greater Mara, it has been suggested that the main drivers of fencing are related to increased livestock densities (livestock biomass within the reserve has increased from 2% of total livestock and wildlife biomass in the 1970s to 23% in the 2000s), wheat and maize cultivation plus human population growth and settlement expansions^19^, sedenterization, climate changes, poaching^18^,^20^ and the reassignment of rights to eliminate any uncertainty arising in the future with regard to how the collective holdings are shared^18^. Between the 1980s and 2000s, these trends led to a more than 70% decline in resident wildlife populations in the Mara^20^,^21^.

## Results

**Figure 2:**
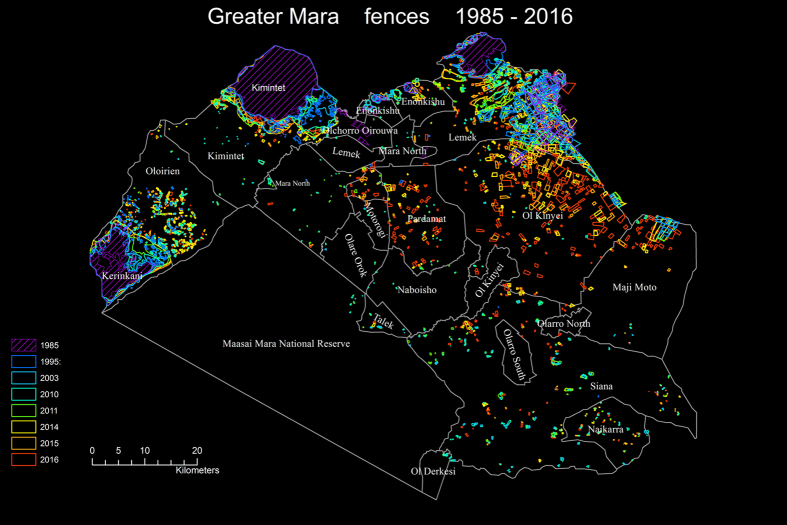
Fences registered on the satellite images (1985–2016). Each year is shown with a distinct color. 1985 is marked with a hatched symbol to emphasize the large, densely fenced areas on the periphery. The figure was created using ArcGIS 10.4.1 ©ESRI. The mapped fences are shown with a distinct colored outline and drawn sequentially with the most recent layers (2016) at bottom, and the oldest (1985) on top. In this way it is recognizable when the individual fences were first observed. The spatial reference is UTM Zone 36S, WGS 1984.

Here, we draw upon a long-term series of Landsat satellite images for mapping fences located within the Greater Mara from 1985 to 2016 (Fig. 2) to quantify the temporal dynamic in areal coverage and geographical distribution of fenced areas in the region.

From 1985 to 2014, fenced plots are concentrated in limited peripheral areas and few in number across the open savannah, exhibiting a slow expansion across this period.

In 2014, fences begin to concentrate in expansion fronts inside the Greater Mara and fill-in areas between these fronts and peripheral concentrations, e.g. in northern Ol Kinyei, and a new front emerges in southern Siana. Furthermore, fences appear in spatial clusters and begin to accumulate in extension of already fenced land plots. In 2016, the front in southern Lemek has radically expanded and fences are now distributed across almost the entire conservancy. Fences have also expanded visibly in the eastern part of Mara North.

**Figure 3:**
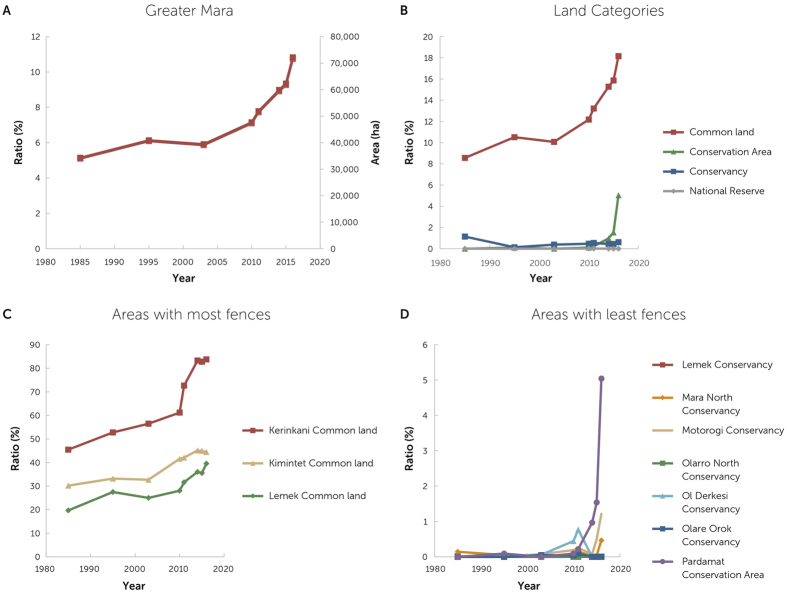
The development in fenced area for the whole Greater Mara as well as for the individual areas. (**A**) Fenced area of the whole Greater Mara in absolute and relative coverage. (**B,C,D**) Percent coverage of fences.

Furthermore, in Ol Kinyei, Lemek and Maji Moto, fences have become far denser in the area between the Greater Mara periphery and the front. In addition, the front surrounding Ol Kinyei has now expanded into the southern part of the Greater Mara. These trends can also be assessed in terms of a percentage of area contained within a fence of the entire area (comprising conservancies, conservation areas and settlement areas) (Fig. 3) showing that, although there are large differences in the policies and practices of the individual community areas, all areas have experienced a strong increase in fencing since 2014.

## Discussion

The period 1985 to 2014 is best described as a phase of ‘availability’. Since 1985 and probably even further back in time, areas on the northern and western outskirts of Mara have been densely fenced. It remains unknown how far back in time these edge concentrations can be followed, but they appear to have been a fairly stable phenomenon within the last 30 years. During this period, there was a relatively slow progression of fences moving out from a few cores of fenced areas along the northern periphery of the Greater Mara, but this movement did not cross borders from one conservancy to the next. Across the open savannah, fenced plots were few in number and little fluctuation can be observed in this period. Until 2014, such fenced plots remained separate and relatively dispersed.

The period 2014 to 2016 is best described as a phase of ‘expansion’ or. Rather than an increased densification of existing stable edge areas, fenced plots begin to pop up and expand across much wider areas of previously open savannah. Numerous new fences have emerged at the edges of conservancies in areas that previously had very few. The stable concentrations at the periphery of the Greater Mara appear to act as hubs from which new fronts are established. Fences are both filling in between the periphery and fronts and expanding out into the open savannah on the other side of the front. Fences from this period are often established in isolation, but in some areas they accumulate in extension of each other. Furthermore, the majority of newly established fenced plots correlate with the established ranches of the area. These new expansion phases of fencing appear as fronts cropping up in southern Lemek and northern Ol Kinyei as well as in scattered locations elsewhere.

### Implications

The rapid acceleration in fencing from 2014 is presently threatening to lead to the collapse of the unique ecosystem of the Greater Mara within a few years. The impending cultural consequences of this loss of area may be that pastoralism and a semi-nomadic lifestyle can no longer be sustained^7^. Ecological consequences already involve the decline of wildlife populations and may also result in the collapse of migrating megafauna populations, with secondary consequences for the rest of the ecosystem^19^,^21^.

In a long-term perspective, the implications may be even more serious. In a historical perspective, comparable processes of large-scale landscape parceling have taken place across other regions and points in history. Although the pace and nature of these fencing dynamics as well as the economic and cultural causes behind them vary, some things are strikingly similar about these processes: (A) they all redefine the relationship between land and people completely, (B) when these processes of land enclosure reach a critical mass, they seem unstoppable; and (C) once boundaries stabilize, they do not generally disappear again^22^,^23^,^24^.

For example, during the early and mid-1^st^ millennium BC, thousands of hectares of land in northern Europe were enclosed and parceled out by so-called ‘Celtic fields’. Although land ownership was subsequently subject to new tenurial systems, these early phases of land enclosure are still visible in modern day heathlands, forests and commons^22^. In Australia and Northern and Southern America, colonization processes in the 18^th^ and 19^th^ century involved claiming land and setting up cattle ranges by means of fences, many of which have also maintained the same position in the landscape to this day^24^,^25^,^26^. These historical scenarios constitute a serious warning that the Greater Mara may be approaching a critical transition to a chronic landscape state shift.

In the Greater Mara, many of the wild herbivores including most of the short-grass grazers such as wildebeest and gazelles are primarily located outside the Maasai Mara National Reserve and in the livestock areas surrounding the conservancies, likely in large part because the grass is shorter there^27^. If fencing increases in these areas adjacent the reserve and the conservancies, then this could have devastating effects on these species. Furthermore, the Greater Mara ecosystem is key to two wildebeest migrations: the Serengeti migration and the Loita plains migration^12^,^28^,^29^,^30^. The main Serengeti migration migrates south into Tanzania to its wet season range in the low-rainfall nutrient-rich Serengeti plains, whereas the smaller Loita plains migration migrates north to the low-rainfall nutrient-rich Loita plains. Since the Greater Mara is a high-rainfall region, it provides reliable forage, and therefore both use the Greater Mara as a dry season range. However, the radically expanding fencing in the central and northeastern part of the Greater Mara is having devastating effects on the Loita plains migration to the far north of the Greater Mara ecosystem, since the fencing there is getting so dense that wildebeest could get completely excluded from this vital migration. Contrary, there is hope that the Maasai Mara Reserve as well as the Mara North conservancy in the south will be able to prevent a similar tragedy. So far, the latter has brought forward local, sustainable management policies that have prevented fences from spreading. These include livestock management programs that manage specific grazing zones and set livestock grazing periods, compensation program in the event of predator-livestock conflicts, moveable and predator-proof enclosures (bomas) to keep livestock safe at night, as well as activities that support natural habitat restoration^31^. Therefore, at present, fencing seems less likely to affect the Serengeti migration. Here, however, another major risk is the planned road that will run across the northern part of the Serengeti National Park, threatening to fraction the main Serengeti migration^32^,^33^,^34^.

### Future research

We anticipate our study to motivate systematic ground-truthing and more sophisticated GIS-based models with a broader geographical coverage and predictive scenarios of the wide-scale, long-term landscape and ecosystem effects of fencing in East Africa. Additionally, we propose that knowledge of comparative past and present land use dynamics is more widely considered to predict the social and economic consequences of expanding fencing and to secure long-term sustainable management^21^,^33^.

The culturally and economically complex and multicausal nature of the fencing phenomenon also means that no simple strategy exists for preventing the remaining areas in the Greater Mara from being fenced, and that interventions in the fencing processes may in some cases cause other problems to arise. Therefore, we suggest a thorough mapping of incentive structures in order to produce a systematic overview of the economic and institutional dimensions of landscape and wildlife management as well as ongoing conservation policies involving changes in fencing practices.

Conservation policies are increasingly putting forward approaches based on polycentric governance and autonomy^34^,^36^,^36^ as well as community-based natural resource management (CBNRM)^34^,^37^,^38^. CBNRM channels social and economic benefits to communities in exchange for their participation in wildlife conservation. Benefits include secured access to land, institutional support, employment, and that profits from ecotourism can be more focused on supporting economically and socially fair and long-lasting partnerships with local communities^39^. We are optimistic that the Greater Mara ecosystem would benefit tremendously from such policies, hereunder focused on the human-animal conflicts directly caused by fencing. For example, elsewhere in Africa, policies are developed in favor of removing fences with direct wish to restore wildlife populations and migrations^40^,^41^. A study of zebra migration in northwest Botswana suggests that, sometimes, migrations can be restored if fences are removed^42^.

In the case of the Greater Mara, this would require the promotion of sustainable grazing as well as wildlife-based economies and incentives to protect and conserve wildlife and communal land, both for conservancies and local private landowners around the Maasai Mara^43^,^44^. Since none of the migration routes are completely within protected areas, it is also vital that communities of people from the areas surrounding the game reserves, including those areas that are now classified as conservation areas, are equally given incentives and resources to protect wildlife and their habitats, including not fencing their land^45^. This is already seen, by many, as a key way to bring income to communities surrounding game reserves and conservancies and could be viable in preventing fences from spreading across the savannahs surrounding the Maasai Mara.

In order to take such steps, and if fencing is to be addressed in a systematic and sustainable way, it is imperative that extensive consultation is made with communities and landowners, conservation organizations, county governments, the Government of Kenya and other stakeholders^45^,^46^,^47^. Importantly, it is imperative that key administrators act now and together and using this vital early warning information to facilitate such initiatives and strategize on the best feasible way to avoid the threatening collapse of the Greater Mara ecosystem.

## Methods

To map the changes in fences located within the Greater Mara, Landsat satellite images between 1985 and 2016 were downloaded (Table 1).

The digitization of fences was done manually based on visual interpretation using ArcMap 10.3.1, by looking at characteristic homogeneous areas using three different color composites as well as a version based on normalized difference vegetation index (NDVI). The three different color composites were (A) red, green and blue (natural), (B) near infrared (NIR), red and green (color infrared/vegetation), (C) short waved infrared (SWIR), NIR and blue (agricultural). In addition to the three compositions and NDVI the contrasts in the images were enhanced to maximum by conducting a principal component transformation of all spectral bands, using the three first components to construct a false color composite images as this enhances the contrasts in both intensity and color hue.

When an assessment of one year was conducted, it was compared to the mapped fences from previous years to ensure that fences from previous years would not be overlooked due to difficulties in detecting them in later years.

To minimize the variation in missed/not-missed-ratio through years, four criteria were used. First, only Landsat satellite images with similar optical sensors and spatial resolution were used. This minimizes the variation in quality of the imagery. Second, only images with a cloud cover of <1% of the area of interest were selected for the study to avoid visibility problems. Third, all images were from mid-January to late-February to minimize the effect from changing seasons. Fourth, the mapping was carried out by the same person in order to minimize the subjectivity of the assessments. Areas, where very high densities of small fences made it impossible to differentiate between individual fences, were digitized as one large compound fence and marked with a hatched symbol. All fences including the compound fences were used for assessing the fenced area coverage.

The fence maps in this study were constructed using visual interpretation of the imagery based on two different contrast enhancements of relevant bands from the visible and near infrared spectrum. The interpretation focused on identifying patterns with straight edges and sharp boundaries. Therefore, we have identified areas exposing a difference between the vegetation within the fenced area as compared to the surrounding landscape matrix. Such vegetation differences take some time to develop, and in many cases with frequent grazing, vegetation inside and outside fences remain almost identical. Therefore, the total amount of fences mapped represents a conservative estimate of the real total.

## Additional Information

**How to cite this article:** Løvschal, M. *et al*. Fencing bodes a rapid collapse of the unique Greater Mara ecosystem. *Sci. Rep.* **7**, 41450; doi: 10.1038/srep41450 (2017).

**Publisher's note:** Springer Nature remains neutral with regard to jurisdictional claims in published maps and institutional affiliations.

## Figures and Tables

**Table 1:** Satellite imagery derived from the USGS download facility^48^.

Year	Date	Satellite	Sensor	Spatial resolution
2016	16 February	Landsat 8	Optical Land Imager	30 m
2015	13 February	Landsat 8	Optical Land Imager	30 m
2014	26 February	Landsat 8	Optical Land Imager	30 m
2011	17 January	Landsat 5	Thematic Mapper	30 m
2010	30 January	Landsat 5	Thematic Mapper	30 m
2003	4 February	Landsat 7	Enhanced Thematic Mapper +	30 m
1995	6 February	Landsat 5	Thematic Mapper	30 m
1985	9 January	Landsat 5	Thematic Mapper	30 m
